# N1 and P1 Components Associate With Visuospatial-Executive and Language Functions in Normosmic Parkinson’s Disease: An Event-Related Potential Study

**DOI:** 10.3389/fnagi.2019.00018

**Published:** 2019-02-05

**Authors:** Yi-Qi Lin, Shi-Shuang Cui, Juan-Juan Du, Gen Li, Yi-Xi He, Ping-Chen Zhang, Yang Fu, Pei Huang, Chao Gao, Bin-Yin Li, Sheng-Di Chen

**Affiliations:** Department of Neurology and Institute of Neurology, Ruijin Hospital Affiliated to Shanghai Jiao Tong University School of Medicine, Shanghai, China

**Keywords:** hyposmia, Parkinson’s disease, event-related potentials, working memory, visuospatial function, language

## Abstract

**Background:** Hyposmia is one of the most important clinical markers of Parkinson’s disease (PD) with a prevalence ranging from 50 to 96% of PD patients. A significant association was found between hyposmia and cognitive impairment of PD. However, there were no reports of event-related potentials (ERP) performance in PD patients with and without hyposmia for cognitive functions assessment.

**Purpose:** The aim of our study was to compare ERP performance and its association with cognitive domains between PD with and without hyposmia.

**Methods:** Olfactory functions were assessed by Sniffin’ Sticks test-16 (SS-16). Twenty-four subjects were included in PD with hyposmia group and nineteen were in PD without hyposmia group. ERP measures were recorded during a delayed match to sample (DMS) task with Chinese characters. The parameters of ERP components including N1, N2, P1, P2, and P3 in retrieval epoch were compared between the two groups and the correlation between ERP results and MOCA item score was also analyzed.

**Results:** No significant difference was found in ERP performance between PD with and without hyposmia. Among all participants, N1 latency was significantly negatively related to visuospatial-executive item score of Montreal Cognitive Assessment (MOCA) (*r*_s_ = -0.381, *P* = 0.012) and P1 amplitude was positively associated with language item score of MOCA (*r*_s_ = 0.302, *P* = 0.049). Within the normosmic group, a significant association was found between N1 latency and visuospatial-executive item score (*r*_s_ = -0.619, *P* = 0.005) and there was also a correlation between language score and P1 amplitude (*r*_s_ = 0.537, *P* = 0.018). In the hyposmic group, only a significant correlation was found between N1 latency and clock drawing test performance (*r*_s_ = -0.413, *P* = 0.045) rather than visuospatial-executive item score. Furthermore, SS-16 score was not found to be significantly associated with either visuospatial-executive or language item score of MOCA.

**Conclusion:** No significant difference was found in ERP components between PD with and without hyposmia. N1 latency and P1 amplitude were respectively associated with visuospatial-executive and language functions in the normosmic group while in the hyposmic group, only a significant correlation was found between N1 latency and clock drawing test performance rather than visuospatial-executive item score in MOCA.

## Introduction

Hyposmia is one of the most important non-motor symptoms of Parkinson’s disease (PD) with a prevalence ranging from 50 to 96% of PD patients (Doty et al., 1988; Wenning et al., 1995; Hawkes et al., 1997; Boesveldt et al., 2008; Duda, 2010). It generally predates motor symptoms and the olfactory testing is useful in differentiating PD from non-PD patients with a sensitivity ranging from 79 to 100% and a specificity from 80 to 89% (Doty, 2012). Therefore, hyposmia is reported to be a critical clinical marker of PD.

In recent years olfactory dysfunction is found to be associated with other non-motor symptoms in PD patients such as chronic constipation, clinical possible rapid eye movement behavior disorder (RBD) (Chen et al., 2015) and psychotic symptoms (Morley et al., 2011). Notably, increasing evidence suggests that hyposmia is strongly associated with cognitive impairment in PD patients and may be a risk factor for PD dementia (Bohnen et al., 2010; Stephenson et al., 2010; Morley et al., 2011; Parrao et al., 2012; Domellof et al., 2017). In fact, the association between olfaction and cognition has been long reported in mild cognitive impairment and Alzheimer’s disease which was evidenced by prominent atrophy in the primary olfactory cortex and hippocampus (Vasavada et al., 2015) as well as a strong correlation between tau pathology in the olfactory bulb and limbic systems (Attems et al., 2005). However, the role of hyposmia in cognitive impairment in PD patients is not clear.

Event-related potentials (ERP) have been widely used for assessing cognitive functions and brain ability. ERP wave latency and amplitude represent respectively the length of time spent and the amount of neural resources participating during information processing. There were increasing studies of ERP in PD patients owning to its independence of motor speed and disability. The abnormal P300 was reported to be associated with cognitive impairment in PD (Katsarou et al., 2004; Matsui et al., 2007) while other ERP measures were barely studied in PD. It remained unclear whether the ERP components were altered in PD with hyposmia, especially the early ERP components reflecting visuospatial processing which was one of the mostly impaired cognitive domains in PD.

The delayed match to sample (DMS) task, one of the most popular tasks during ERP records in PD patients (Seer et al., 2016), was used in our study to test working memory which related to several cognitive abilities including storage capacity, retrieval strategies (Unsworth and Engle, 2007) and visuospatial attention (Bleckley et al., 2003; Giuliano et al., 2014). And Chinese characters applied in our DMS task were more acceptable for Chinese participants and more dependable on visual working memory (Opitz et al., 2014). What’s more, we focused on retrieval epoch rather than encoding epoch in view of impaired retrieval process and reserved encoding ability of memory deficit in PD (Mahurin et al., 1993).

To our knowledge, there was no research focusing on cognitive ERP measures in PD patients with hyposmia. The aim of this study was to improve our understanding of the role of hyposmia in cognitive impairment in PD using the ERP technique with DMS task. Our study investigated difference in ERP components and its association with cognitive domains between PD patients with and without hyposmia.

## Materials and Methods

### Subject

Idiopathic PD patients aging from 50 to 80 were recruited from movement disorder clinic of the Department of Neurology, Ruijin Hospital affiliated to Shanghai Jiao Tong University School of Medicine from September 2016 to October 2017. PD was diagnosed according to the clinical Movement Disorder Society (MDS) diagnostic criteria (Postuma et al., 2015) by senior Movement Disorder Specialist. Exclusion criteria included history of head injury, stroke, psychiatric disorder, poor vision, nasal and paranasal disease or other factors affecting olfactory function. Patients with less than 6 years of education or Mini-Mental State Examination (MMSE) score less than 24 were also excluded.

Finally 43 PD patients were enrolled in our study. Hyposmia was defined with score of Sniffin’ Sticks test-16 (SS-16) (Burghart Messtechnik, Wedel, Germany) less than 8.3 (Chen et al., 2012) which was consistent with previous researches in our department (Chen et al., 2015). There were 24 subjects in PD with hyposmia group and 19 in PD without hyposmia group. All participants were informed of the research protocol and this study was carried out in accordance with the recommendations of Ethical Review of Biomedical Research Involving Human Subjects by China’s Ministry of Health with written informed consent from all subjects. The protocol was approved by Ethics Committee of Ruijin Hospital affiliated to Shanghai Jiao Tong University School of Medicine, Shanghai, China.

### Neuropsychological Assessment

Demographic characters including age, sex, education years and disease duration were recorded (Table 1). The disease progression was assessed by modified Hoehn and Yahr (H-Y) scale (Hoehn and Yahr, 1967) and levodopa equivalent dose (LED) which was calculated as reported (Tomlinson et al., 2010). Motor subtypes were divided into tremor dominant type and non-tremor dominant type. Montreal Cognitive Assessment (MOCA) Beijing version was used to evaluate cognitive performance (Yu et al., 2012). Night sleep quality was assessed by Parkinson’s disease Sleep Scale (PDSS) (Wang et al., 2008). Rapid Eye Movement Sleep Behavior Disorder Questionnaire-Hong Kong (RBD-HK) (Shen et al., 2014) and Epworth Sleepiness Scale (ESS) (Kumar et al., 2003) were used to evaluate RBD and excessive daytime sleepiness (EDS). The anxiety symptoms were evaluated by Hamilton Anxiety Rating Scale (HAMA) (Thompson, 2015) and 17-item Hamilton Rating Scale for Depression (HAMD-17) (Hamilton, 1960) was used for depression screening.

**Table 1:** Demographics and clinical characteristics in PD with and without hyposmia groups (mean ± standard deviation).

	PD without hyposmia (*n* = 19)	PD with hyposmia (*n* = 24)	*P*
Age (years)	62.37 ± 6.19	64.71 ± 5.89	0.213
Sex (M/*n*)	8/19	11/24	0.807
Disease duration (years)	5.31 ± 5.46	5.63 ± 4.29	0.835
LED (mg)	269.66 ± 260.57	280.65 ± 174.76	0.871
Education (years)	12.05 ± 2.30	12.92 ± 2.50	0.250
H-Y stage	1.68 ± 0.71	1.75 ± 0.59	0.742
Motor subtype (tremor type/*n*)	7/19	11/22	0.553
SS-16	10.58 ± 1.64	6.00 ± 2.02	0.000
PDSS	126.84 ± 20.61	117.75 ± 16.59	0.116
RBD-HK	14.84 ± 17.58	13.13 ± 14.00	0.723
ESS	5.95 ± 4.76	5.71 ± 4.84	0.872
HAMD-17	3.74 ± 3.96	3.83 ± 4.06	0.938
HAMA	5.16 ± 4.80	3.75 ± 3.18	0.255
MOCA	25.79 ± 1.93	26.79 ± 2.64	0.173
MMSE	28.68 ± 1.46	28.46 ± 2.06	0.688


### The Delayed Match to Sample Task

**FIGURE 1:**
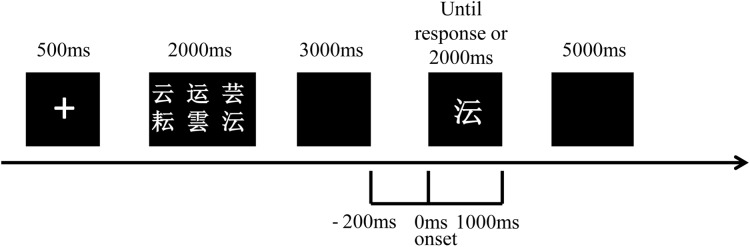
Schematic representation of the visual DMS task used for ERP recordings. A set of six Chinese characters (sample stimulus) was displayed after a single of “+” and was required to be remembered by participants. After a blank for 3000 ms, a character (probe stimulus) presented for 2000 ms and the answer of “yes” or “no” was required to decide whether this character was a member of the set of characters or not. The inter-trial interval was 5000 ms. The retrieval period is from 200 ms before probe stimulus onset to 1000 ms after.

Each participant was required to participate in a practice block before the test block during the DMS task (Figure 1). In each trial, after a single of “+” in the screen of 500 ms, a set of six visually similar and easily confused Chinese characters (sample stimulus) presented for 2000 ms which should be remembered by subjects. After a blank for 3000 ms, a character (probe stimulus) demonstrated in the middle of the screen could either be a member of the previous set of characters (answer “yes”) or be another similar-looking one (answer “no”). The character presented until a response before 2000 ms or until 2000 ms without response. Subjects were asked to answer “yes” by pressing the button of “1” or “no” by pressing the button of “2” on a keyboard as soon as the response was ensured. The “yes” and “no” answers were randomized and 50% of the correct answers were “yes” and the rest was “no” in each block. The inter-trial interval was 5000 ms. Patients were allowed to begin the test until the accuracy of the practice block was higher than 50%. Each test contained 100 trials. This DMS task was programmed by E-Prime 2.0 software (Psychology Software Tools, Inc., Pittsburgh, PA, United States) in which reaction time and accuracy of each subject were also recorded. The retrieval period of our study was defined from 200 ms before probe stimulus onset to 1000 ms after.

### EEG Recordings and Data Analysis

ERP was recorded from 32 Ag-AgCl electrodes (Fp1, Fp2, F3, F4, C3, C4, P3, P4, O1, O2, F7, F8, T7, T8, P7, P8, Fz, FCz, Cz, Pz, FC1, FC2, CP1, CP2, FC5, FC6, CP5, CP6, FT9, FT10, TP9, TP10) which were placed according to the international 10–20 system with a 32-channel amplifier (BrainAmp by Brain Products, Munich, Germany).

All channels were digitized at 500 Hz with 200 ms-long prestimulus baseline used for baseline correction. The filter band-pass was 0.1–70 Hz. Electro-oculogram (EOG) was also recorded to correct ocular artifacts. A semi-automatic check was used in artifact rejection. Trials were rejected if EEG voltage step was higher than 50 μV/ms or if difference of values in intervals was higher than 200 μV or lower than 0.5 μV. The mean rejection rate for all participants was higher than 1%. ERPs were based on correct trials and data not rejected for artifacts.

The five ERP components including N1, N2, P1, P2, and P300 were analyzed in reference of early studies, meanwhile, electrodes with maximal amplitude were chosen, which was consistent with the previous report of our center using the same paradigm (Li et al., 2016). N1 amplitude was measured as the maximal negative peak within the latency window of 150–210 ms on P7, P8 electrodes. P1 amplitude was identified at the most positive peak on O1, O2, P7 and P8 within 84–140 ms after the stimulus onset. N2 amplitude was considered at the maximal negative peak on F3, F4, C3, C4, CZ, FZ and FCz within the latency window of 230–300 ms. P2 amplitude was identified at the maximal positive peak on FC1, FC2, CZ, FZ and FCz within the latency window of 150–250 ms. The P300component amplitude was analyzed as the maximal positive peak within 250–450 ms on O1, O2 and Pz electrodes. The N1P2 amplitude was summed absolute amplitude of the N1 and P2 peaks on P7, P8, FC1, FC2, CZ, FZ and FCz electrodes. The N1P2 amplitude divided by average noise level yielded the signal-to-noise (S/N) ratio.

### Statistical Analysis

All statistical analysis was performed by SPSS 19.0 (IBM, Armonk, NY). Demographic characteristics and clinical variables were compared between the two groups using the independent sample *t*-test or chi square analysis. The Spearman’s correlation was analyzed between each ERP component parameter and MOCA item score. The association between SS-16 and MOCA item score was also analyzed by Spearman’s correlation. All ERP components parameters were compared between the two groups using independent Mann–Whitney *U*-test. All tests were 2-tailed and *P*-values < 0.05 were considered statistically significant.

## Results

### Demographics and Clinical Characteristics

There was no significant group difference in demographic characters including age, sex, and education degree. No significant difference was revealed in disease duration, H-Y stage, tremor subtype and LED between PD with and without hyposmia. Questionnaire results of sleep disorder (PDSS, RBD-HK and ESS) as well as anxiety (HAMA) and depression (HAMD-17) were similar between the two groups. No significant difference was found in total score of MMSE or MOCA (Table 1).

### Task Performance and Brain Activity

**FIGURE 2:**
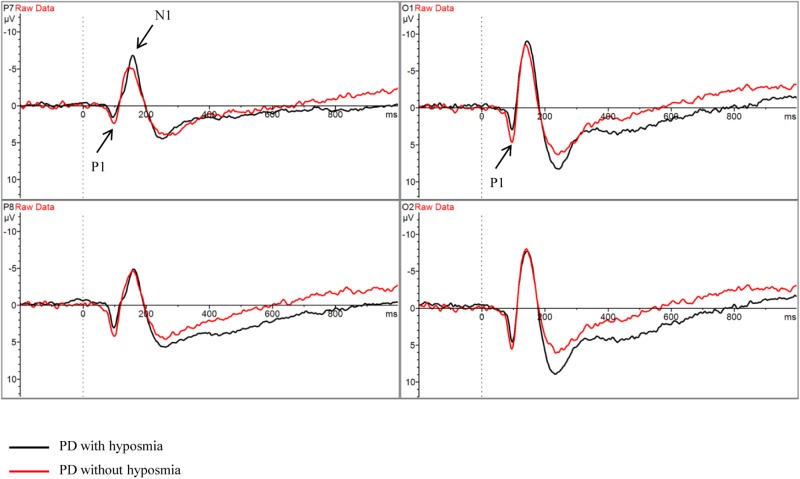
Grand average ERP waveform in PD with hyposmia (black line) and PD without hyposmia (red line) during retrieval period in DMS task. Latency was recorded in milliseconds with stimulus onset at 0. Amplitude was recorded in micro-voltage.

There was no significant difference between PD patients with and without hyposmia regarding number of valid trials, reaction time and accuracy. No significant difference was found in parameters of ERP components including N1, N2, P1, P2 and P300 between the two groups (Table 2 and Figure 2). Notably, the N1P2 amplitude did not differ between the two groups. And the mean S/N ratio was 4.90 in the normosmic group and 5.17 in the hyposmic group and no difference was found between the two groups (Table 2).

**Table 2:** Task performance and parameters of ERP components in PD with and without hyposmia groups (mean ± standard deviation).

	PD without hyposmia (*n* = 19)	PD with hyposmia (*n* = 24)	*P*
Number of valid trials	1625	2048	0.954
Accuracy (%)	89.79 ± 8.38	88.21 ± 19.39	0.742
Reaction time (s)	1590.49 ± 226.51	1549.82 ± 246.54	0.581
N1 latency (ms)	162.16 ± 12.05	158.54 ± 7.75	0.265
N1 amplitude (μV)	-6.05 ± 3.30	-7.17 ± 3.92	0.391
N2 latency (ms)	261.98 ± 15.06	262.42 ± 21.12	0.940
N2amplitude (μV)	-1.94 ± 3.29	-1.89 ± 2.88	0.964
P1 latency (ms)	98.58 ± 8.00	99.83 ± 9.30	0.643
P1 amplitude (μV)	4.98 ± 3.31	4.77 ± 2.75	0.818
P2 latency (ms)	169.43 ± 20.04	173.35 ± 28.51	0.615
P2 amplitude (μV)	5.39 ± 3.97	5.63 ± 2.63	0.807
P3 latency (ms)	372.25 ± 54.80	391.53 ± 46.93	0.221
P3 amplitude (μV)	5.03 ± 2.96	6.40 ± 4.01	0.220
N1P2 amplitude (μV)	8.28 ± 4.83	8.23 ± 3.19	0.509
S/N	4.90 ± 2.32	5.17 ± 2.53	0.826


### Correlation Between ERPs and Cognitive Ability

Correlation was analyzed between ERP component parameter and cognitive performance in all participants (Tables 3, 4). N1 latency was significantly negatively related to visuospatial-executive item score of MOCA (*r*_s_ = -0.381, *P* = 0.012). Among three visuospatial-executive tests, a significant negative correlation between N1 latency and test score was found in cube copy test (*r*_s_ = -0.401, *P* = 0.008) and clock drawing (*r*_s_ = -0.451, *P* = 0.002) but not in trail making test. What’s more, a significant positive correlation was found between P1 amplitude and language item score of MOCA (*r*_s_ = 0.302, *P* = 0.049). There was no correlation between MOCA total score and N1 or P1 parameters. Parameters of other ERP components including N2, P2 and P300 were not found significantly associated with MOCA score or MOCA item score. Then the correlations between N1 latency and P1 amplitude and visuospatial-executive and language item score were also analyzed in separate groups (Table 5). There was a significant negative correlation between N1 latency and visuospatial-executive item score within the normosmic group (*r*_s_ = -0.619, *P* = 0.005) but not within the hyposmic group. Among three visuospatial tests, a significant negative correlation with N1 latency was found in both cube copy test (*r*_s_ = -0.585, *P* = 0.008) and clock drawing test (*r*_s_ = -0.523, *P* = 0.022) within the normosmic group and only in clock drawing test (*r*_s_ = -0.413, *P* = 0.045) within the hyposmic group. In addition, a significant correlation between P1 amplitude and language item score was found within the normosmic group (*r*_s_ = 0.537, *P* = 0.018) but not within the hyposmic group. Furthermore, SS-16 score was not found to be significantly associated with visuospatial-executive or language item score of MOCA (Table 6).

**Table 3:** Correlation analysis between ERP latency and MOCA score in all participants (correlation coefficient *r*_s_).

	N1	N2	P1	P2	P3
Visuospatial-executive	-0.381*	0.085	-0.068	-0.104	0.121
Visuospatial-executive (trail making test)	-0.105	0.107	-0.067	0.103	0.079
Visuospatial-executive (cube copy)	-0.401**	0.178	-0.009	0.000	0.094
Visuospatial-executive (clock drawing)	-0.451**	0.021	-0.037	-0.207	0.069
Naming	-0.044	0.084	-0.038	-0.191	0.164
Attention	0.092	-0.131	0.000	0.041	-0.023
Language	0.101	-0.027	0.036	0.020	0.237
Abstraction	-0.232	-0.125	0.051	-0.088	0.193
Delayed Recall	0.034	0.199	-0.003	0.136	0.236
Orientation	-0.206	0.080	-0.004	-0.067	-0.098
Total score	-0.217	0.100	-0.024	-0.003	0.223


*^∗^P < 0.05; ^∗∗^P < 0.01.*

**Table 4:** Correlation analysis between ERP amplitude and MOCA score in all participants (correlation coefficient *r*_s_).

	N1	N2	P1	P2	P3
Visuospatial-executive	0.010	-0.077	-0.185	-0.288	-0.147
Visuospatial-executive (trail making test)	-0.100	-0.079	-0.234	-0.188	-0.205
Visuospatial-executive (cube copy)	0.053	0.193	-0.094	-0.164	-0.228
Visuospatial-executive (clock drawing)	0.035	-0.067	-0.018	-0.179	-0.016
Naming	0.059	-0.206	0.193	0.037	0.200
Attention	0.053	0.003	-0.161	-0.034	0.081
Language	-0.195	-0.120	0.302*	0.231	0.075
Abstraction	-0.140	-0.069	-0.033	-0.038	0.222
Delayed Recall	0.142	0.226	-0.114	0.088	0.040
Orientation	-0.214	0.071	-0.089	0.169	-0.169
Total score	0.005	0.089	-0.053	0.020	0.002


*^∗^P < 0.05.*

**Table 5:** Correlation analysis between parameters of ERP components (N1 and P1) and MOCA item (visuospatial-executive and language items) score in PD with and without hyposmia groups (correlation coefficient *r*_s_).

	PD without hyposmia		PD with hyposmia	
		
	N1 latency	P1 amplitude	N1 latency	P1 amplitude
Visuospatial- executive	-0.619**	-0.182	-0.161	-0.121
Visuospatial- executive (trail making test)	-0.303	-0.207	0.119	-0.153
Visuospatial- executive (cube copy)	-0.585**	-0.184	-0.153	0.044
Visuospatial- Executive (clock drawing)	-0.523*	0.040	-0.413*	-0.059
Language	0.104	0.537*	0.098	0.032


*^∗^P < 0.05; ^∗∗^P < 0.01.*

**Table 6:** Correlation analysis between SS-16 score and MOCA item (visuospatial-executive and language items) score in all participants (correlation coefficient *r*_s_).

	SS-16
Visuospatial-executive	-0.088
Visuospatial-executive (trail making test)	-0.034
Visuospatial-executive (cube copy)	-0.118
Visuospatial-executive (clock drawing)	-0.125
Language	-0.201
Total score	-0.099


Correlation was also analyzed between parameters of N1 and P1 components and clinical characteristics in all subjects (Table 7). N1 latency was found to be significantly negatively related to education degree (*r*_s_ = -0.326, *P* = 0.033). There was a significant positive correlation between HAMA score and N1 amplitude (*r*_s_ = 0.360, *P* = 0.018). No significant correlation was found between other component parameters and clinical characteristics.

**Table 7:** Correlation analysis between parameters of ERP components (N1 and P1) and clinical characteristics in all participants (correlation coefficient *r*_s_).

	N1 latency	N1 amplitude	P1 latency	P1 amplitude
Age (years)	-0.177	0.143	0.058	0.175
Disease duration (years)	0.037	-0.105	0.203	0.076
Education degree (years)	-0.326*	-0.091	-0.232	-0.058
LED (mg)	0.086	0.028	0.162	0.134
ESS	0.089	0.041	0.287	0.086
PDSS	0.260	-0.128	-0.046	0.226
RBD-HK	-0.047	0.163	0.003	0.191
H-Y stage	0.091	0.149	0.236	0.051
HAMA	0.127	0.360*	0.035	0.047
HAMD-17	-0.074	0.191	-0.045	-0.095


*^∗^P < 0.05.*

## Discussion

To our knowledge, our study was the first to compare cognitive ERP measures between PD patients with and without hyposmia. No significant difference in ERP performance was found between the two groups. Among all subjects, N1 latency was significantly negatively related to visuospatial-executive function and there was a significant positive correlation between P1 amplitude and language ability. Within the normosmic group, N1 and P1 also associated with visuospatial-executive and language functions while a significant correlation was only found between N1 latency and clock drawing test performance rather than visuospatial-executive item score in the hyposmic group. What’s more, odor identification ability was not found to be significantly related to visuospatial-executive function or language ability.

Hyposmia is an important clinical biomarker of PD and there are four subtypes of olfactory dysfunction reported in PD including impairment of odor identification, odor discrimination, odor threshold detection and odor recognition memory (Mesholam et al., 1998; Haehner et al., 2009). Odor identification deficit is the most prevalent form of hyposmia and is tested mainly by University of Pennsylvania Smell Identification Test (UPSIT) in PD patients (Doty et al., 1988) and SS-16 (Haehner et al., 2009) which was used in our study considering its advantage in cross-cultural application.

Previous studies found a significant association between hyposmia and cognitive impairment in PD patients. The percentage of cognitive decline was significantly higher in PD patients with hyposmia than those without (Bohnen et al., 2010; Stephenson et al., 2010; Morley et al., 2011; Parrao et al., 2012). And olfactory dysfunction significantly increased the risk of dementia in PD patients regardless the cognitive functions at baseline according to a 10-year follow up study (Domellof et al., 2017). The underlying mechanism remained unclear. The olfactory bulb was known to be one of the onset sites with appearance of Lewy body pathology during PD progression in Braak’s stage (Braak et al., 2003). And over expression of alpha-synuclein in the olfactory bulb was reported to initiate hyposmia and other prodromal symptoms of PD in rats (Niu et al., 2018). A hypothesis was proposed that severe olfactory dysfunction might be related to abundant cortical Lewy body deposition (Morley et al., 2011) which was found to be associated with PD dementia (Tsuboi et al., 2007). What’s more, the relation between hyposmia and cognitive impairment could also be explained by cholinergic denervation of the limbic archicortex (Bohnen et al., 2010) and dopaminergic denervation of the hippocampus (Bohnen et al., 2008). In our study, no significant difference was found in MMSE or MOCA score between PD with and without hyposmia, which may be associated with limits of our sample size and sensitivities of MMSE and MOCA in cognitive impairment of PD patients (Burdick et al., 2014; Wyman-Chick et al., 2017). Interestingly, no significant difference was found in parameters of ERP components including N1, N2, P1, P2 and P300 recorded during DMS tasks between these two groups. Therefore, the association between cognitive domains and ERP measures in PD with and without hyposmia was important to be investigated.

N1 and P1 were found to respectively associate with visuospatial-executive functions and language in our study while no significant correlation was found between the other components including P2, N2, and P300 and specific cognitive functions. N1 and P1 are the earliest components in ERP representing visual sensory input. The generator of visual N1 is probably located in lateral extrastriate cortex (Gomez Gonzalez et al., 1994), dorsal occipito-parietal and ventral occipito-temporal areas (Yamazaki et al., 2000) and the visual P1 may be generated in fusiform gyrus (Heinze et al., 1994). N1 and P1 were reported to be associated with visual perceptual processing (Vogel and Luck, 2000; Taylor, 2002) especially in visuospatial attention according to previous reports (Naatanen and Picton, 1987; Luck et al., 1990). Luck et al. (1990) found that N1 was related to attention orienting to relevant stimulus and P1 possibly reflected a facilitation of early sensory processing with attention already focused. On the other hand, several studies demonstrated the role of N1 and P1 in language perception. Selpien et al. (2015) found that N1 and P1 were significantly related to language lateralization. In an auditory ERP study, the association between P1 amplitude and attention was found in linguistic probes stimulation but not in non-linguistic probes (Giuliano et al., 2014).

Abnormal N1 and P1 components in PD patients were observed in several studies. Wright et al. (1996) found attenuated N1 amplitudes during Auditory Oddball Task in 17 PD patients. However, enlarged N1 and P1 amplitudes as well as shortened N1 latency in 34 PD patients were demonstrated in a visual ERP study with oddball and S1–S2 tasks (Li et al., 2003). The discrepancy may be explained by the differences of tasks used in ERP, sample size and analytical approach of component parameters. Unfortunately, the clinical significance of N1 and P1 components in PD patients was investigated in very few studies. Wang et al. (2001) reported the association between N1 and cognitive visual processing in PD patients evidenced by its correlation with the regional cerebral blood flow. Consistent to previous studies, our study found a significant negative correlation between N1 latency and visuospatial-executive function in PD patients and P1 amplitude was significantly positively related to language ability. Therefore N1 latency and P1 amplitude during DMS task were possible to be used for assessing visuospatial-executive functions and language abilities in PD patients. Interestingly, the significant association between these cognitive functions and N1 and P1 components were also found in the normosmic group while in the hyposmic group, there was only a significant correlation between N1 and clock drawing test score rather than visuospatial-executive item score. To explain difference in clinical significance of N1 and P1 components between the two groups, one hypothesis could be that there exists different cognitive processing between these normosmic and hyposmic participants. Increasing evidence shows that PD with and without hyposmia may be two subtypes of PD patients. Lee et al. (2015) found that normosmic PD represented a unique clinical phenotype with a more benign with fewer motor deficits and higher dopamine transporter activity. On the other hand, hyposmia in PD was reported to be associated with tremor-dominant type (Iijima et al., 2011), SNCA rs11931074 and specific non-motor symptoms such as RBD and chronic constipation (Chen et al., 2015). From the aspect of cognition, a good olfactory performance in PD was found to compensate gray matter volume loss (Lee et al., 2014) and there were cholinergic and dopaminergic denervations in PD with hyposmia (Bohnen et al., 2008, 2010). Therefore, some components underlying a cognitive process such as visuospatial-executive and language functions could be different between PD with and without hyposmia.

The impaired cognitive domains in PD with hyposmia were under debate. Bohnen et al. (2010) found that UPSIT scores were significantly related to episodic verbal learning in 58 PD patients but not other cognitive performance including visuospatial function, visual non-verbal memory, attention and executive function. However, poorer memory and executive functions were reported to be associated with worse olfactory identification in other studies (Morley et al., 2011; Parrao et al., 2012). Our study demonstrated no significant association between SS-16 score and visuospatial-executive or language item score of MOCA in PD patients. The discrepancy is possibly explained by difference in sample size and neurocognitive test batteries which may be avoided by using ERP measures.

There were several limitations of our study. A larger sample size is expected in further studies to confirm our results. In addition, only MOCA with relatively high reliability and validation reported (Nie et al., 2012) was used for cognitive assessment in our study as the psychometric properties of neurocognitive test batteries were rarely studied in Chinese PD patients. What’s more, only odor identification was tested which was the mostly impaired olfactory function in PD (Doty et al., 1988). However, in view of possibly different mechanisms underlying four subtypes of hyposmia (Kareken et al., 2003; Hedner et al., 2010), the association between cognitive domains and the other three olfactory functions in PD warrants further investigation.

In conclusion, our study compared ERP measures recorded during DMS tasks at retrieval period and its association with cognitive domains between PD patients with and without hyposmia. No significant difference in ERP components was found between the two groups in the present study. Among all participants, N1 latency was significantly negatively related to visuospatial-executive functions and there was also a significantly positive correlation between P1 amplitude and language ability. In PD without hyposmia, N1 latency and P1 amplitude were respectively associated with visuospatial-executive and language functions while in PD with hyposmia, only a significant correlation was found between N1 latency and clock drawing test performance rather than visuospatial-executive item score in MOCA. What’s more, no significant association was found between odor identification ability and visuospatial-executive or language item score of MOCA.

## Author Contributions

All the authors cooperated and contributed to the design and plan of the present study. Y-QL, S-SC, J-JD, GL, Y-XH, P-CZ, YF, PH, and CG performed the research. S-DC was in charge of clinical diagnosis. Y-QL and B-YL analyzed the data. Y-QL wrote the original article. S-DC and B-YL were in charge of manuscript verifying.

## Conflict of Interest Statement

The authors declare that the research was conducted in the absence of any commercial or financial relationships that could be construed as a potential conflict of interest.
